# End-of-Life Care for Older Adults With Dementia by Race and Ethnicity and Physicians’ Role

**DOI:** 10.1001/jamahealthforum.2025.4235

**Published:** 2025-11-14

**Authors:** Deborah M. Oyeyemi, Ryo Ikesu, Debra Saliba, Anne M. Walling, Utibe R. Essien, Keith C. Norris, Alexandra Klomhaus, Haiyong Xu, Hiroshi Gotanda, Yusuke Tsugawa

**Affiliations:** 1 Division of General Internal Medicine and Health Services Research, Department of Medicine, David Geffen School of Medicine at UCLA, Los Angeles, California; 2 National Clinician Scholars Program at UCLA, Los Angeles, California; 3 Department of Epidemiology, UCLA Fielding School of Public Health, Los Angeles, California; 4 Geriatric Research, Education, and Clinical Centers, Veterans Affairs Greater Los Angeles Healthcare System, Los Angeles, California; 5 UCLA Borun Center, Los Angeles, California; 6 RAND Corporation, Santa Monica, California; 7 Center for the Study of Healthcare Innovation, Implementation, and Policy, Veterans Affairs Greater Los Angeles Healthcare System, Los Angeles, California; 8 Department of Medicine Statistics Core, David Geffen School of Medicine at UCLA, Los Angeles, California; 9 Division of General Internal Medicine, Cedars-Sinai Medical Center, Los Angeles, California; 10 Department of Health Policy and Management, UCLA Fielding School of Public Health, Los Angeles, California

## Abstract

**Importance:**

Evidence is limited regarding whether end-of-life care for individuals with dementia varies by race and ethnicity, and whether observed variations can be explained by differences in the physicians providing their care.

**Objective:**

To evaluate end-of-life care among individuals with dementia across racial and ethnic groups, and to investigate whether care variations are explained by differences in treating physicians.

**Design, Setting, and Participants:**

This cohort study used national, population-based claims data from a 20% random sample of Medicare fee-for-service beneficiaries aged 66 years or older with a diagnosis of dementia who died between 2016 and 2019. Data were analyzed from January 2024 to June 2025.

**Main Outcomes and Measures:**

Emergency department, hospital, and intensive care unit use, mechanical ventilation or cardiopulmonary resuscitation, and feeding tube placement in last 30 days of life; death in acute care hospital; hospice use and palliative care counseling in last 180 days of life; and any billed advance care planning before death.

**Results:**

Among 259 945 decedents with dementia (mean [SD] age, 85.8 [8.0] years; 60.4% female), 8.3% were non-Hispanic Black, 4.4% were Hispanic, and 87.3% were non-Hispanic White. Compared with non-Hispanic White decedents, non-Hispanic Black decedents were more likely to receive emergency department (difference, 5.7 percentage points [pp]; 95% CI, 5.0-6.4 pp), hospital (difference, 4.0 pp; 95% CI, 3.3-4.7 pp), intensive care unit (difference, 4.3 pp; 95% CI, 3.7-4.9 pp), mechanical ventilation or cardiopulmonary resuscitation (difference, 3.8 pp; 95% CI, 3.3-4.3 pp), and feeding tube placement (difference, 1.8 pp; 95% CI, 1.5-2.1 pp) care, as well as die in a hospital (difference, 3.5 pp; 95% CI, 2.9-4.1 pp). Non-Hispanic Black decedents were less likely to use hospice (difference, −6.1 pp; 95% CI, −6.8 to −5.4 pp) and more likely to receive palliative care counseling (difference, 3.2 pp; 95% CI, 2.6-3.9 pp) and billed advance care planning (difference, 1.8 pp; 95% CI, 1.2-2.3 pp) than non-Hispanic White decedents. Similar patterns were observed among Hispanic decedents. Variations in end-of-life care remained qualitatively unchanged when comparing decedents treated by the same physician.

**Conclusions and Relevance:**

Findings from this cohort study suggest that non-Hispanic Black and Hispanic decedents with dementia received more intensive end-of-life care despite higher rates of billed advance care planning and palliative care counseling than non-Hispanic White decedents. Observed racial and ethnic variations were not explained by differences in the physicians treating them.

## Key Points

**Question:**

Does end-of-life care for individuals with dementia vary by race and ethnicity, and can these variations be explained by differences in the physicians providing their care?

**Findings:**

In this cohort study of 259 945 decedents with dementia, non-Hispanic Black and Hispanic decedents were less likely to use hospice and more likely to receive billed advance care planning, palliative care counseling, and intensive end-of-life care than non-Hispanic White decedents. Variations in end-of-life care utilization by race and ethnicity persisted when comparing decedents treated by the same physician.

**Meaning:**

These findings suggest that end-of-life care utilization varies by race and ethnicity among decedents with dementia, but these variations are not explained by differences in the physicians providing their care.

## Teaser

This cohort study evaluates whether end-of-life care utilization among individuals with dementia varies by race and ethnicity and physicians’ role in treatment.

## Introduction

Nearly 7 million older adults in the US have Alzheimer disease, the most common cause of dementia.^1,2^ Dementia is a progressive, neurodegenerative condition associated with increased morbidity and mortality.^1,3,4,5^ End-of-life care utilization and processes associated with dementia may vary by race and ethnicity of patients.^6,7^

Black and Hispanic individuals with dementia, in particular, are more likely to receive intensive end-of-life care.^7,8,9,10,11,12^ Among individuals with dementia, rates of emergency department use,^8^ hospitalizations,^7,8,9^ intensive care unit admission,^7^ feeding tube placement,^10,11^ and death in the hospital^7,12^ are higher among Black and Hispanic individuals. In addition, studies have reported lower advance care planning completion rates and less palliative care and hospice use among these groups.^8,13,14,15,16^ Although it is challenging to infer potential differences in care context or patients’ care preferences in these studies,^17^ the extent of racial and ethnic variations in end-of-life care utilization raises questions about the quality of care older adults with dementia receive. These care differences also have financial implications. A 2022 study by Lin et al^8^ found that non-Hispanic Black and Hispanic older adults with dementia had approximately 60% higher Medicare inpatient expenditures in the last few months of life than their non-Hispanic White counterparts. Higher utilization of end-of-life care services may lead to increased economic burden among Black and Hispanic older adults with dementia and their families.^18,19,20^

Although racial and ethnic variations in end-of-life care have been previously studied,^7,8,9,10,11,12,13,14,15,16^ the underlying mechanisms causing these differences remain largely unknown. In addition to structural and social factors including variable access to services such as palliative care and hospice, timeliness of dementia diagnoses, and differences in cultural values and care preferences,^13,21,22^ one potential contributing factor is the role of physicians’ practice patterns. Physicians participate in advance care planning discussions and counsel patients on palliative care and hospice use.^23,24,25^ Further, studies have found significant variations in physicians’ practice patterns and suggest that non-Hispanic Black and Hispanic patients may seek and receive care from lower-performing physicians compared with non-Hispanic White patients.^26,27,28,29^ These differences in physician performance may be attributed to multiple factors, including limited access to high-quality hospitals and physicians as well as a pervasive mistrust of health care professionals among these communities.^30,31,32^ Many dementia studies do not consider physicians in their end-of-life care analyses; therefore, we included physician-level variation in our comprehensive analysis of end-of-life care among older adults with dementia from different racial and ethnic groups.

In this study, we used Medicare claims data to evaluate end-of-life care utilization and processes for individuals with dementia by race and ethnicity, and to determine whether observed variations could be explained by differences in the physicians from whom they receive care.

## Methods

### Data and Study Population

This retrospective cohort study was conducted from January 2024 to July 2025 and followed Strengthening the Reporting of Observational Studies in Epidemiology (STROBE) reporting guidelines. The Institutional Review Board at the University of California, Los Angeles reviewed the study and waived informed consent because decedent data were deidentified. We used a 20% random sample of Medicare fee-for-service (FFS) claims data from 2016 through 2019. The initial sample was restricted to beneficiaries aged 66 years or older who had died on or later than July 1, 2016, had continuous enrollment in Medicare Parts A and B, and had a claims-based diagnosis of Alzheimer disease and Alzheimer disease–related dementias (AD/ADRD).^33^ For Medicare beneficiaries to be assigned a diagnosis of dementia in the Chronic Conditions Data Warehouse (CCW), they must have at least 1 inpatient, skilled nursing facility, home health, Part B institutional, or Part B noninstitutional (carrier) claim with a related code during the 3-year reference period.

A previous study validated that 82% of clinically diagnosed cases of dementia had relevant *International Classification of Disease*, *Ninth Revision* codes in Medicare claims with 3 years of follow-up.^34^ Among Medicare FFS decedents with AD/ADRD diagnoses from 2016 through 2019, we further limited the sample to decedents who had a practicing primary care physician. We identified evaluation and management (E&M) claims for primary care services filed by physicians with primary care specialties (family practice, general practice, internal medicine, or geriatric medicine) and attributed each beneficiary to a primary care physician who billed 50% or more of the E&M claims for the beneficiary in the last 6 months of life (eMethods and eTable 1 in Supplement 1).^35,36,37^ We excluded beneficiaries if we could not attribute them to a physician, for example, when beneficiaries did not have any E&M claims in the last 6 months.

Racial and ethnic identity was derived from the Medicare–Research Triangle Institute race and ethnicity variable (eMethods in Supplement 1).^38,39^ Non-Hispanic Black (hereafter Black), Hispanic, and non-Hispanic White (hereafter White) decedents were included in the study. Individuals from other racial and ethnic groups (eg, American Indian and Alaska Native, Asian, and Native Hawaiian or Other Pacific Islander) were excluded due to small sample sizes.

### End-of-Life Care Utilization and Process Measures

 We used 9 binary end-of-life care utilization and process measures (a list of codes is provided in eTable 2 in Supplement 1): (1) any emergency department visit in the last 30 days of life; (2) any hospitalization in the last 30 days of life; (3) any intensive care unit visit in the last 30 days of life; (4) mechanical ventilation, cardiopulmonary resuscitation (CPR), or defibrillation in the last 30 days of life; (5) feeding tube placement in the last 30 days of life; (6) death in an acute care hospital without hospice; (7) hospice use in the last 180 days of life; (8) palliative care counseling in the last 180 days of life; and (9) any billed advance care planning before death.

### Adjustment Variables

We adjusted for decedent characteristics, including age (66-75 [reference category], 76-85, 86-95, and 96 years or older), sex, median household income in the last 12 months (quartiles), continuous Medicaid coverage during the last 6 months of life, 25 CCW comorbidities (27 CCW conditions minus 2 dementia-related diagnoses),^33^ nursing home residence (defined as having a comprehensive or quarterly Minimum Data Set assessment within 90 days of death^40^), and month and year of death. Median household income estimates were obtained by residential zip code in 2019 inflation-adjusted US dollars using data from the American Community Survey.^41^

### Statistical Analysis

We compared each of the 9 end-of-life care measures by the decedents’ racial and ethnic identity using 2 regression models. The first regression model adjusted for decedent characteristics and hospital referral region fixed effects to account for differences in practice patterns within a geographical area. In the second regression model, we also adjusted for primary care physician fixed effects to account for physician-level variation, effectively comparing outcomes among patients treated by the same physician. After fitting each regression model, we used the marginal standardization method to calculate adjusted outcome rates. Specifically, we estimated the probabilities of outcomes for each patient averaged over the distribution of covariates in our national sample, fixing the indicator variable for racial and ethnic identity at each level.^42^

To address the possibility that physicians who were defined as primary care physicians in our approach may include physicians in non–office-based settings (eg, skilled nursing facilities) because many individuals may have been in institutional settings in the last 6 months of life, we conducted 2 sensitivity analyses using alternative attribution algorithms. First, we attributed a beneficiary to a primary care physician based solely on office-based E&M claims.^37^ Second, we used E&M claims from the last 12 months of life rather than the last 6 months. Additionally, we repeated the analysis by using claims from office and outpatient settings to define palliative care counseling and billed advance care planning, because beneficiaries in institutional settings (ie, skilled nursing facilities and hospitals) may have received palliative care counseling and advance care planning billed in those settings by physicians who are not their actual primary care physician. Finally, to address the possibility that Black and Hispanic beneficiaries, despite their higher risk for dementia, may have a lower likelihood of having dementia diagnosed compared with White beneficiaries, ^1,43,44^ we conducted a sensitivity analysis after limiting the sample to Medicare beneficiaries who received annual wellness visits (AWVs). Given that the Medicare AWV requires direct cognitive assessment as one of its components, and prior studies have found that dementia diagnosis increased due to AWVs,^45,46,47^ we hypothesized that the difference in dementia diagnosis by race and ethnicity was smaller for this population.

We also conducted secondary analyses to evaluate whether racial and ethnic variations in end-of-life care utilization and processes changed over time. In these analyses, we estimated the first regression model (ie, without physician fixed effects) including a continuous year variable, instead of a categorical variable, and interaction terms between the continuous year variable and the racial and ethnic identity variable. We then formally tested the coefficients of the interaction terms, which represented the annual changes of the outcomes of interest.

Statistical analyses were conducted using SAS, version 9.4 (SAS Institute Inc) and Stata MP, version 16.1 (StataCorp LLC). Statistical tests were 2-tailed, with *P* < .05 indicating statistical significance.

## Results

### Beneficiary Characteristics

Among 259 945 decedents with dementia, 8.3% were Black, 4.4% were Hispanic, and 87.3% were White (Table 1). Mean (SD) decedent age was 85.8 (8.0) years; on average, White decedents with dementia were older than Black and Hispanic decedents. Overall, 60.4% of all decedents were female and 39.6% were male. Compared with White decedents with dementia, Black and Hispanic decedents had lower median household incomes and were a greater proportion of Medicaid beneficiaries. Fewer Hispanic decedents were nursing home residents (44.9%) compared with Black (53.8%) and White (50.0%) decedents. Black and Hispanic decedents had higher prevalence of chronic kidney disease, diabetes, and heart failure, and Black decedents had higher cancer prevalence when compared with the overall cohort (Table 1).

**Table 1.** Descriptive Characteristics for Dementia Decedents by Race and Ethnicity

Characteristic	%			
	Overall (N = 259 945)	Non-Hispanic Black (n = 21 678)	Hispanic (n = 11 368)	Non-Hispanic White (n = 226 899)
Age, mean (SD), y	85.8 (8.0)	83.5 (8.8)	84.7 (8.2)	86.1 (7.9)
Sex				
Female	60.4	60.4	58.7	60.5
Male	39.6	39.6	41.3	39.5
Median household income, $ (SD)^a^	67 238.83 (27 898.33)	54 668.51 (24 623.09)	60 839.55 (25 490.56)	68 760.42 (27 959.51)
Medicaid coverage	29.0	48.0	59.5	25.6
Nursing home residence	50.1	53.8	44.9	50.0
Selected comorbidities				
Cancer^b^	14.8	17.7	11.9	14.7
Chronic kidney disease	62.4	77.7	71.4	60.5
COPD	29.4	28.9	30.3	29.4
Diabetes	39.9	59.6	60.4	37.0
Heart failure	54.2	59.2	57.1	53.6

Abbreviation: COPD, chronic obstructive pulmonary disease.

^a^ Median household income in the past 12 months (in 2019 inflation-adjusted US dollars) estimated from residential zip code.

^b^ Cancer includes individuals with breast, colorectal, endometrial, lung, or prostate cancer.

### End-of-Life Care Utilization and Processes by Race and Ethnicity

We found racial and ethnic variations across each of the 9 end-of-life care measures when adjusting for only beneficiary characteristics and hospital referral region fixed effects (ie, without physician fixed effects) (Table 2). Black and Hispanic decedents with dementia had more intensive end-of-life care than White decedents, with increased likelihood of an emergency department visit (Black decedents: difference, 5.7 percentage points [pp]; 95% CI, 5.0-6.4 pp; *P* < .001; Hispanic decedents: difference, 5.1 pp; 95% CI, 4.1-6.0 pp; *P* < .001), hospitalization (Black decedents: difference, 4.0 pp; 95% CI, 3.3-4.7 pp; *P* < .001; Hispanic decedents: difference, 5.5 pp; 95% CI, 4.6-6.5 pp; *P* < .001), and an intensive care unit visit (Black decedents: difference, 4.3 pp; 95% CI, 3.7-4.9 pp; *P* < .001; Hispanic decedents: difference, 5.3 pp; 95% CI, 4.4-6.2 pp; *P* < .001) in the last 30 days of life. Compared with White decedents, Black and Hispanic decedents were also more likely to receive mechanical ventilation or CPR (Black decedents: difference, 3.8 pp; 95% CI, 3.3-4.3 pp; *P* < .001; Hispanic decedents: difference, 3.2 pp; 95% CI, 2.5-4.0 pp; *P* < .001) and have a feeding tube placed (Black decedents: difference, 1.8 pp; 95% CI, 1.5-2.1 pp; *P* < .001; Hispanic decedents: difference, 0.5 pp; 95% CI, 0.2-0.8 pp; *P* = .001). Black and Hispanic decedent groups were more likely to die in an acute care hospital (Black decedents: difference, 3.5 pp; 95% CI, 2.9-4.1 pp; *P* < .001; Hispanic decedents: difference, 3.4 pp; 95% CI, 2.6-4.2 pp; *P* < .001) than White decedents.

**Table 2.** End-of-Life Care Utilization and Processes for Dementia Decedents by Race and Ethnicity

Outcome by race and ethnicity	Models without physician fixed effects^a^			Models with physician fixed effects^a^		
	Adjusted probability, % (95% CI)	Adjusted probability difference, percentage point (95% CI)	*P* value	Adjusted probability, % (95% CI)	Adjusted probability difference, percentage point (95% CI)	*P* value
**ED visit in last 30 d of life**						
Non-Hispanic White	52.8 (52.5 to 53.0)	0 [Reference]	NA	52.8 (52.7 to 52.9)	0 [Reference]	NA
Non-Hispanic Black	58.4 (57.8 to 59.1)	5.7 (5.0 to 6.4)	<.001	58.3 (57.5 to 59.1)	5.5 (4.7 to 6.4)	<.001
Hispanic	57.8 (56.9 to 58.7)	5.1 (4.1 to 6.0)	<.001	57.0 (55.8 to 58.1)	4.1 (3.0 to 5.3)	<.001
**Hospitalization in last 30 d of life**						
Non-Hispanic White	46.5 (46.3 to 46.7)	0 [Reference]	NA	46.6 (46.5 to 46.7)	0 [Reference]	NA
Non-Hispanic Black	50.5 (49.8 to 51.1)	4.0 (3.3 to 4.7)	<.001	50.2 (49.4 to 51.0)	3.6 (2.8 to 4.5)	<.001
Hispanic	52.0 (51.1 to 52.9)	5.5 (4.6 to 6.5)	<.001	50.9 (49.8 to 52.0)	4.3 (3.2 to 5.5)	<.001
**ICU visit in last 30 d of life**						
Non-Hispanic White	23.1 (22.9 to 23.3)	0 [Reference]	NA	23.3 (23.2 to 23.4)	0 [Reference]	NA
Non-Hispanic Black	27.4 (26.8 to 28.0)	4.3 (3.7 to 4.9)	<.001	26.6 (25.9 to 27.3)	3.4 (2.6 to 4.1)	<.001
Hispanic	28.4 (27.6 to 29.3)	5.3 (4.4 to 6.2)	<.001	27.0 (26.0 to 28.1)	3.8 (2.7 to 4.9)	<.001
**Mechanical ventilation or cardiopulmonary resuscitation in last 30 d of life**						
Non-Hispanic White	10.5 (10.4 to 10.6)	0 [Reference]	NA	10.6 (10.5 to 10.7)	0 [Reference]	NA
Non-Hispanic Black	14.3 (13.8 to 14.8)	3.8 (3.3 to 4.3)	<.001	13.5 (12.9 to 14.1)	2.9 (2.3 to 3.6)	<.001
Hispanic	13.7 (13.0 to 14.4)	3.2 (2.5 to 4.0)	<.001	13.1 (12.3 to 14.0)	2.5 (1.6 to 3.4)	<.001
**Feeding tube placement in last 30 d of life**						
Non-Hispanic White	1.3 (1.2 to 1.3)	0 [Reference]	NA	1.3 (1.3 to 1.3)	0 [Reference]	NA
Non-Hispanic Black	3.1 (2.8 to 3.3)	1.8 (1.5 to 2.1)	<.001	2.9 (2.6 to 3.2)	1.6 (1.3 to 1.9)	<.001
Hispanic	1.8 (1.5 to 2.1)	0.5 (0.2 to 0.8)	.001	1.8 (1.4 to 2.1)	0.5 (0.1 to 0.8)	.03
**Death in acute care hospital**						
Non-Hispanic White	15.9 (15.7 to 16.0)	0 [Reference]	NA	16.0 (15.9 to 16.1)	0 [Reference]	NA
Non-Hispanic Black	19.4 (18.8 to 19.9)	3.5 (2.9 to 4.1)	<.001	18.9 (18.2 to 19.6)	2.9 (2.2 to 3.6)	<.001
Hispanic	19.3 (18.5 to 20.1)	3.4 (2.6 to 4.2)	<.001	17.7 (16.8 to 18.7)	1.7 (0.7 to 2.7)	.001
**Hospice use in last 180 d of life**						
Non-Hispanic White	61.5 (61.3 to 61.7)	0 [Reference]	NA	61.4 (61.3 to 61.5)	0 [Reference]	NA
Non-Hispanic Black	55.4 (54.7 to 56.1)	−6.1 (−6.8 to −5.4)	<.001	55.2 (54.3 to 56.0)	−6.3 (−7.2 to −5.3)	<.001
Hispanic	59.5 (58.6 to 60.5)	−1.9 (−2.9 to −0.9)	<.001	61.0 (59.8 to 62.1)	−0.4 (−1.7 to 0.7)	.48
**Palliative care counseling in last 180 d of life**						
Non-Hispanic White	19.9 (19.7 to 20.0)	0 [Reference]	NA	20.0 (19.9 to 20.1)	0 [Reference]	NA
Non-Hispanic Black	23.1 (22.5 to 23.7)	3.2 (2.6 to 3.9)	<.001	21.6 (21.0 to 22.3)	1.6 (0.9 to 2.3)	<.001
Hispanic	21.2 (20.4 to 21.9)	1.3 (0.5 to 2.1)	.001	21.4 (20.5 to 22.4)	1.4 (0.4 to 2.4)	.004
**Billed advance care planning before death**						
Non-Hispanic White	16.4 (16.3 to 16.6)	0 [Reference]	NA	16.5 (16.4 to 16.5)	0 [Reference]	NA
Non-Hispanic Black	18.2 (17.6 to 18.7)	1.8 (1.2 to 2.3)	<.001	17.7 (17.1 to 18.3)	1.2 (0.6 to 1.9)	<.001
Hispanic	17.7 (16.9 to 18.5)	1.3 (0.5 to 2.1)	.002	17.7 (16.8 to 18.6)	1.2 (0.3 to 2.1)	.007

Abbreviations: ED, emergency department; ICU, intensive care unit; NA, not applicable.

^a^ Each model adjusted for age, sex, Medicaid coverage, nursing home residence, household income as estimated from residential zip code, comorbidities, year and month of death, and hospital referral region fixed effects. Model with physician fixed effects also adjusted for physician-level variation, effectively comparing beneficiaries treated by the same physician.

By contrast, Black and Hispanic decedents with dementia were less likely to receive hospice (Black decedents: difference, −6.1 pp; 95% CI, −6.8 to −5.4 pp; *P* < .001; Hispanic decedents: difference, −1.9 pp; 95% CI, −2.9 to −0.9 pp; *P* < .001) despite being more likely to receive palliative care counseling (Black decedents: difference, 3.2 pp; 95% CI, 2.6-3.9 pp; *P* < .001; Hispanic decedents: difference, 1.3 pp; 95% CI, 0.5-2.1 pp; *P* = .001) than their White counterparts. In addition, billed advance care planning was greater for Black and Hispanic decedents at 18.2% and 17.7%, respectively, compared with White decedents (Black decedents: difference, 1.8 pp; 95% CI, 1.2-2.3 pp; *P* < .001; Hispanic decedents: difference, 1.3 pp; 95% CI, 0.5-2.1 pp; *P* = .002).

Adjustment for physician fixed effects (effectively comparing treatment by the same physician) led to variable changes in magnitude for some outcome variables but no qualitative change in our overall findings (Table 2). For example, when we compared variations between Hispanic and White decedents, differences in hospitalization, intensive care unit admission, mechanical ventilation or CPR, and death in an acute care hospital decreased by more than 20% after adjustment for physician fixed effects, indicating that only 20% of the variations could be explained by differences in physicians who treated them. When we compared variations between Black and White decedents, differences similarly decreased by more than 20% for intensive care unit admission and mechanical ventilation or CPR. Differences in hospice use between Black and White decedents (difference, −6.3 pp; 95% CI, −7.2 to −5.3 pp; *P* < .001) and in palliative care counseling between Hispanic and White decedents (difference, 1.4 pp; 95% CI, 0.4-2.4 pp; *P* = .004) widened after adjustment for physician fixed effects. There was only 1 measure for which adjustment for physician fixed effects eliminated racial and ethnic variation; difference in hospice use between Hispanic and White decedents was no longer significant after adjusting for physician fixed effects (difference, −0.4 pp; 95% CI, −1.7 to 0.7 pp; *P* = .48).

### Sensitivity Analyses

The results did not change qualitatively when we attributed a beneficiary to a PCP based solely on office-based E&M claims or when we used E&M claims from the last 12 months of life rather than the last 6 months (eTables 3 and 4 in Supplement 1). In the analysis using claims from office and outpatient settings to define palliative care counseling and billed advance care planning, we found no evidence that the likelihood of receiving palliative care counseling and billed advance care planning in office and outpatient settings differed by race and ethnicity of decedents (eTable 5 in Supplement 1). The sensitivity analysis among Medicare beneficiaries who received AWV yielded results similar to the main analysis, although the estimates were less precise (eTable 6 in Supplement 1).

### Trends in End-of-Life Care Utilization and Processes by Race and Ethnicity

In the secondary analyses, we assessed trends of each end-of-life care measure during the study period to determine whether variations changed over time from 2016 through 2019 (Figure; eTable 7 in Supplement 1). For billed advance care planning, the annual percentage-point increase was greater for Black decedents (difference, 1.8 pp change; 95% CI, 1.3-2.3; *P* < .001 for interaction) and Hispanic decedents (difference, 1.1 pp change; 95% CI, 0.5-1.8; *P* = .001 for interaction) compared with White decedents with dementia, indicating that differences in advance care planning between Black and Hispanic decedents and their White counterparts widened during this period. Regarding palliative care counseling, the annual percentage-point increase was also greater for Black decedents (difference, 0.6 pp change; 95% CI, 0.0-1.1; *P* = .04 for interaction), suggesting a widening of the difference between Black and White decedents. Conversely, annual differences for all other end-of-life care measures did not significantly change between Black and White decedents or Hispanic and White decedents during the study period, indicating that racial and ethnic variations for most end-of-life care outcomes were generally stable from 2016 through 2019.

**Figure.**
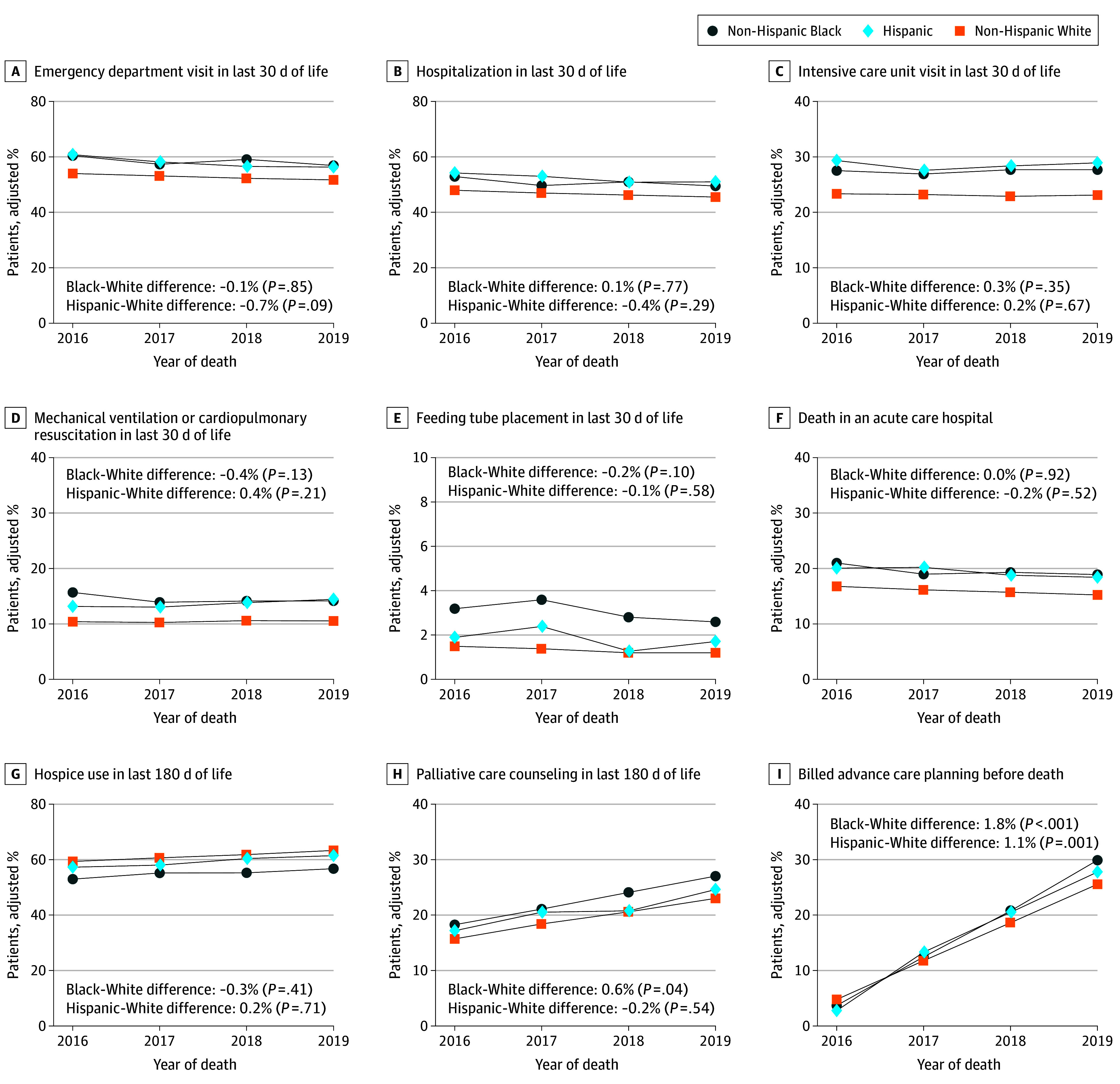
Trends in End-of-Life Care Utilization and Processes for Dementia Decedents by Race and Ethnicity, 2016-2019 Each model adjusted for age, sex, Medicaid coverage, nursing home residence, household income as estimated from residential zip code, comorbidities, month and year of death, and hospital referral region fixed effects. Differences between Black and White decedents (Black-White difference) and Hispanic and White decedents (Hispanic-White difference) represent respective differences in average annual change for each outcome measure, with a positive coefficient indicating a widening difference and a negative coefficient indicating a narrowing difference over time. *P* value represents the interaction of decedent race and ethnicity with year of death in the adjusted model, with White patients as the reference group.

## Discussion

In this cohort study of Medicare data, we found that end-of-life care among decedents with dementia varied by race and ethnicity. Although Black and Hispanic decedents with dementia had a higher likelihood of receiving advance care planning and palliative care counseling, they experienced more intensive end-of-life care than White decedents. These findings remained largely unchanged when comparing patients treated by the same physician. Overall, our findings suggest that racial and ethnic variations in end-of-life care could not generally be explained by variations in treating physicians.

We found that the observed racial and ethnic variations in end-of-life care among individuals with dementia did not narrow much when we compared patients treated by the same physician, suggesting that these variations may not be explained by differences in access to physicians who provide high-quality end-of-life care. Instead, our findings suggest the potential influence of other factors, such as cultural values and care preferences, which may contribute to more intensive care at the end of life among Black and Hispanic decedents with dementia.^8,48,49^ These possibilities underscore the need for multifaceted approaches that address the social and structural barriers in health care. Such approaches are essential to ensure timely dementia diagnosis, optimal counseling on disease progression and treatment options, and adherence to patient and family goals and preferences throughout the trajectory of dementia for all racial and ethnic groups.^50^ Future research is warranted to understand the underlying mechanisms causing these racial and ethnic variations in end-of-life care patterns.

We found that Black and Hispanic decedents with dementia did not have lower prevalence of billed advance care planning and palliative care counseling compared with their White counterparts, contrary to the findings in prior literature. In addition, we found no evidence that the likelihood of receiving palliative care counseling and billed advance care planning in office and outpatient settings differed by race and ethnicity of decedents. These findings indicate that the higher likelihood of receiving palliative care counseling and billed advanced care planning among Black and Hispanic decedents is probably due to their intensive care at the end of life in institutional settings. It is important to note that our study assessed billed advance care planning, which only indicates that a discussion of the patient’s health care wishes took place and does not require the completion of an advance directive. In contrast, previous studies have relied on informant interviews to assess advance care planning completion.^8^ The discrepancy between our findings on advance care planning and palliative care and those of prior studies suggests evolving trends toward more palliative care and advance care planning for older adults with dementia, especially among Black and Hispanic individuals.

Despite having increased advance care planning and palliative care counseling, Black and Hispanic decedents experienced more intensive end-of-life care. Emergency department visits, hospitalizations, and intensive care unit visits were among the end-of-life care areas with greatest variations for Black and Hispanic decedents. These findings were consistent with previous studies that did not adjust for physician fixed effects.^7,8,9^ It is possible that variations in emergency department visits and hospitalizations were partly due to racial and ethnic differences in care preferences.^22^ Some studies have noted less preference for comfort care among Black and Hispanic older adults with dementia and their caregivers, possibly due to factors like cultural views, religious preferences, and prior experiences with health care.^8,48,49^

### Limitations

This study has some limitations. First, with respect to the study sample, we excluded older adults who lacked a claims-based dementia diagnosis, continuous Medicare enrollment, or a primary care physician. These sampling constraints likely led to the exclusion of some of the most vulnerable beneficiaries.^51^ Second, health care utilization was measured using the Medicare FFS claims, and these claims may not fully capture the entirety of care rendered to individuals with dementia. For example, palliative care counseling or advance care planning discussions that were not billed by physicians were not observed. Additionally, Black and Hispanic beneficiaries may be less likely to be able to afford health care services, especially those in institutional settings, potentially leading to an underestimation of their health care service utilization evaluated with claims data. Third, our study was unable to determine whether high-intensity care at the end of life was concordant with patients’ care preferences. However, a previous survey-based study conducted among Medicare beneficiaries has suggested that people generally prefer treatments focused on symptom palliation rather than life extension.^52^ Fourth, our analysis accounted for variation at the level of the primary physician, but multiple health care professionals can play a role in decedents’ care. Fifth, our study focused on FFS or traditional Medicare beneficiaries. Approximately half of all Medicare beneficiaries were enrolled in Medicare Advantage (MA) plans in 2024, compared with 33% to 39% during the study period.^53,54^ Medicare FFS and MA plans differ with respect to payment incentives and risk adjustment.^55^ Because we did not have access to data on beneficiaries enrolled in MA plans, this study focused on Medicare FFS beneficiaries. Although MA beneficiaries present an opportunity for future inquiry, we believe that this study’s unique perspective on physicians’ contribution to variation in end-of-life care has important implications for the dementia population.

## Conclusions

In this cohort study, end-of-life care utilization and processes among older adult Medicare beneficiaries with dementia varied by race and ethnicity, even when comparing patients treated by the same physician. Our findings extend existing research by demonstrating that these care variations persist despite accounting for physician-level variation and underscore the need to explore other factors, such as structural and social barriers to care, for their potential role in driving more intensive end-of-life care utilization among Black and Hispanic older adults with dementia.

## Notes

Supplement 1.
eMethods
**eTable 1.** Billing codes used to attribute a beneficiary to a primary care physician **eTable 2.** CPT, ICD-10, and other codes used to identify end-of-life care utilization and processes in Medicare claims, 2016-2019 **eTable 3.** End-of-life care utilization and processes for dementia decedents by race and ethnicity, using office-based E&M claims to attribute a beneficiary to a primary care physician **eTable 4.** End-of-life care utilization and processes for dementia decedents by race and ethnicity, using E&M claims from the last 12 months of life to attribute a beneficiary to a primary care physician **eTable 5.** End-of-life care utilization and processes for dementia decedents by race and ethnicity, using claims from office and outpatient settings to define palliative care counseling and billed advance care planning **eTable 6.** End-of-life care utilization and processes for dementia decedents with annual wellness visits by race and ethnicity **eTable 7.** Changes in end-of-life care utilization and processes for dementia decedents by race and ethnicity, 2016-2019
eReferences

Supplement 2.
Data Sharing Statement
