# Supplementary material for: End-of-Life Care for Older Adults With Dementia by Race and Ethnicity and Physicians’ Role
Source: JAMA Health Forum. 2025 Nov 14;6(11):e254235. doi: 10.1001/jamahealthforum.2025.4235 (PMC12619101; doi:10.1001/jamahealthforum.2025.4235)
Supplement: Supplement 1. — eMethods eTable 1. Billing codes used to attribute a beneficiary to a primary care physician eTable 2. CPT, ICD-10, and other codes used to identify end-of-life care utilization and processes in Medicare claims, 2016-2019 eTable 3. End-of-life care utilization and processes for dementia decedents by race and ethnicity, using office-based E&M claims to attribute a beneficiary to a primary care physician eTable 4. End-of-life care utilization and processes for dementia decedents by race and ethnicity, using E&M claims from the last 12 months of life to attribute a beneficiary to a primary care physician eTable 5. End-of-life care utilization and processes for dementia decedents by race and ethnicity, using claims from office and outpatient settings to define palliative care counseling and billed advance care planning eTable 6. End-of-life care utilization and processes for dementia decedents with annual wellness visits by race and ethnicity eTable 7. Changes in end-of-life care utilization and processes for dementia decedents by race and ethnicity, 2016-2019 eReferences [file jamahealthforum-e254235-s001.pdf]

Supplementary Online Content

Oyeyemi DM, Ikesu R, Saliba D, et al. End-of-life care for older adults with dementia by race and ethnicity and physicians' role. *JAMA Health Forum*. Published online November 14, 2025. doi:10.1001/jamahealthforum.2025.4235

eMethods
eTable 1. Billing codes used to attribute a beneficiary to a primary care physician
eTable 2. CPT, ICD-10, and other codes used to identify end-of-life care utilization and processes in Medicare claims, 2016-2019
eTable 3. End-of-life care utilization and processes for dementia decedents by race and ethnicity, using office-based E&M claims to attribute a beneficiary to a primary care physician
eTable 4. End-of-life care utilization and processes for dementia decedents by race and ethnicity, using E&M claims from the last 12 months of life to attribute a beneficiary to a primary care physician
eTable 5. End-of-life care utilization and processes for dementia decedents by race and ethnicity, using claims from office and outpatient settings to define palliative care counseling and billed advance care planning
eTable 6. End-of-life care utilization and processes for dementia decedents with annual wellness visits by race and ethnicity
eTable 7. Changes in end-of-life care utilization and processes for dementia decedents by race and ethnicity, 2016-2019
eReferences

This supplemental material has been provided by the authors to give readers additional information about their work.

© 2025 Oyeyemi DM et al. JAMA Health Forum.

eMethods

Attribution of Primary Care Physicians

Among Medicare Fee-for-Service (FFS) decedents with Alzheimer's disease and Alzheimer's disease-related dementias (AD/ADRD) diagnoses from 2016-2019, we further limited the sample to decedents who had a practicing primary care physician. We identified evaluation and management (E&M) claims for primary care services filed by physicians with primary care specialties (family practice, general practice, internal medicine, or geriatric medicine) and attributed each beneficiary to a "primary care physician," based on the number of E&M claims for the beneficiary in the last six months of life (**Supplementary Table 1**). "Plurality" of E&M claims (i.e., attributing patients to a provider who accounted for the largest number of E&M claims) is used by the Centers for Medicare & Medicaid Services (CMS) to attribute Medicare beneficiaries to Accountable Care Organizations, and this approach has also been used widely in prior research.[1–3] We used a stricter definition by restricting to those physicians who billed 50% or more of the E&M claims. This narrower definition avoids attributing patients to physicians when multiple physicians are taking care of patients and it is unclear who the primary physician is. For example, if the physician who billed the largest number of E&M claims accounted for 35% of total claims, while another physician billed 30%, it would be unclear which physician is primarily responsible for the patient's end-of-life care if we used the standard plurality criteria. We excluded inpatient E&M claims in order to exclude hospitalist physicians from our sample of primary care physicians. We excluded beneficiaries if we could not attribute them to a physician, for example, when beneficiaries did not have any E&M claims in the last six months.

© 2025 Oyeyemi DM et al. JAMA Health Forum.

Identification of Race and Ethnicity

Racial and ethnic identity was derived from the Medicare-Research Triangle Institute (RTI) race and ethnicity variable. The RTI race and ethnicity variable was developed to improve the identification of Hispanic and Asian or Pacific Islander individuals by combining Social Security Administration (SSA) race codes with probabilities of each race and ethnicity group derived from beneficiaries' first and last names.[4,5] This algorithm enhances the accuracy of race and ethnicity classification, identifying more individuals as Hispanic or Asian or Pacific Islander compared to relying solely on the SSA race codes. For Hispanic individuals, the RTI algorithm increases sensitivity from 29.5% to 76.6% without a loss of specificity.[5]

© 2025 Oyeyemi DM et al. JAMA Health Forum.

eTable 1. Billing Codes Used to Attribute a Beneficiary to a Primary Care Physician

Current Procedural Terminology codes	Description
99201-99205 & 99211-99215	Office or other outpatient services
99304-99310 & 99315-99316 & 99318	Nursing facility services
99324-99328 & 99334-99337	Domiciliary, rest home, or custodial care services
99339-99340	Domiciliary, rest home, or home care plan oversight services
99341-99345 & 99347-99350	Home services
G0402	Initial preventive exam
G0438	Annual wellness visit, initial visit
G0439	Annual wellness visit, subsequent visit
G0463	Hospital outpatient clinic visit for assessment and management of a patient

© 2025 Oyeyemi DM et al. JAMA Health Forum.

eTable 2. CPT, ICD-10, and Other Codes Used to Identify End-of-Life Care Utilization and Processes in Medicare Claims, 2016-2019

Study outcomes	Codes
Emergency department visit	Any claim with REV_CNTR = 0450–0459, 0981
Hospitalization	NCH_BENE_DSCHRG_DT in Inpatient file for PRVDR_NUM= (3rd digit =0) or (3rd and 4th digit =13) (only acute care or critical access hospitals)
Intensive care unit visit	REV_CNTR = 0200-0209 in Inpatient file
Mechanical ventilation	ICD-10 codes: 5A1935Z, 5A1945Z, 5A1955Z, 5A09357, 5A09358, 5A0935Z, 5A09457, 5A09458, 5A0945Z, 5A09557, 5A09558, 5A0955Z
Cardiopulmonary resuscitation and/or defibrillation	ICD-10 codes: 5A12012, 5A19054, 5A2204Z
Feeding tube placement	CPT codes: 43246, 43653, 43830, 43832, 44300, 44372, 49440, 49441<br>ICD-10 codes: 0DH64UZ, 0DH63UZ, 0DW04UZ, 0DW08UZ
Hospice use	CLM_FROM_DT in Hospice file
Palliative care counseling	ICD-10 code: Z51.5
Billed advance care planning	CPT codes: 99497, 99498

© 2025 Oyeyemi DM et al. JAMA Health Forum.

eTable 3. End-of-Life Care Utilization and Processes for Dementia Decedents by Race and Ethnicity, Using Office-Based E&M Claims to Attribute a Beneficiary to a Primary Care Physician

Outcomes	Race and ethnicity	Models without physician fixed effects			Models with physician fixed effects		
		Adjusted probability, % (95% CI)	Adjusted probability difference, pp (95% CI)	P-value	Adjusted probability, % (95% CI)	Adjusted probability difference, pp (95% CI)	P-value
ED visit in last 30 days of life	NH White	63.6 (63.4 to 63.9)	Ref	Ref	63.7 (63.6 to 63.9)	Ref	Ref
	NH Black	67.2 (66.4 to 68.1)	+3.6 (+2.7 to +4.5)	<0.001	66.4 (65.1 to 67.7)	+2.7 (+1.3 to +4.1)	<0.001
	Hispanic	67.6 (66.4 to 68.8)	+4.0 (+2.8 to +5.3)	<0.001	67.6 (65.8 to 69.4)	+3.9 (+2.0 to +5.8)	<0.001
Hospitalization in last 30 days of life	NH White	57.2 (56.9 to 57.5)	Ref	Ref	57.4 (57.3 to 57.6)	Ref	Ref
	NH Black	58.9 (58.0 to 59.8)	+1.7 (+0.8 to +2.6)	<0.001	57.3 (56.0 to 58.5)	-0.2 (-1.6 to +1.3)	0.83
	Hispanic	61.9 (60.7 to 63.1)	+4.7 (+3.4 to +5.9)	<0.001	60.8 (58.9 to 62.6)	+3.4 (+1.4 to +5.3)	0.001
ICU visit in last 30 days of life	NH White	29.2 (28.9 to 29.4)	Ref	Ref	29.3 (29.1 to 29.4)	Ref	Ref
	NH Black	32.0 (31.2 to 32.9)	+2.9 (+2.0 to +3.8)	<0.001	31.1 (29.8 to 32.3)	+1.8 (+0.4 to +3.1)	0.01
	Hispanic	34.1 (32.9 to 35.4)	+5.0 (+3.7 to +6.2)	<0.001	33.5 (31.7 to 35.2)	+4.2 (+2.3 to +6.0)	<0.001
Mechanical ventilation or cardiopulmonary resuscitation in last 30 days of life	NH White	13.5 (13.3 to 13.7)	Ref	Ref	13.5 (13.4 to 13.7)	Ref	Ref
	NH Black	17.1 (16.4 to 17.9)	+3.7 (+2.9 to +4.4)	<0.001	16.2 (15.1 to 17.2)	+2.6 (+1.5 to +3.8)	<0.001
	Hispanic	16.4 (15.3 to 17.4)	+2.9 (+1.8 to +3.9)	<0.001	16.5 (15.0 to 18.0)	+2.9 (+1.4 to +4.5)	<0.001
Feeding tube placement in last 30 days of life	NH White	1.5 (1.5 to 1.6)	Ref	Ref	1.5 (1.5 to 1.6)	Ref	Ref
	NH Black	3.5 (3.1 to 3.9)	+2.0 (+1.6 to +2.4)	<0.001	3.6 (3.1 to 4.1)	+2.1 (+1.5 to +2.6)	<0.001
	Hispanic	2.0 (1.6 to 2.4)	+0.4	0.04	2.0 (1.4 to 2.5)	+0.4	0.14

© 2025 Oyeyemi DM et al. JAMA Health Forum.

			(+0.0 to +0.9)			(-0.1 to +1.0)	
Death in acute care hospital	NH White	20.3 (20.1 to 20.6)	Ref	Ref	20.6 (20.4 to 20.7)	Ref	Ref
	NH Black	23.4 (22.6 to 24.3)	+3.1 (+2.2 to +4.0)	<0.001	22.0 (20.8 to 23.2)	+1.4 (+0.1 to +2.7)	0.03
	Hispanic	23.6 (22.4 to 24.7)	+3.2 (+2.1 to +4.4)	<0.001	21.7 (20.0 to 23.3)	+1.1 (-0.6 to +2.8)	0.22
Hospice use in last 180 days of life	NH White	60.1 (59.8 to 60.4)	Ref	Ref	59.9 (59.8 to 60.1)	Ref	Ref
	NH Black	52.8 (51.9 to 53.8)	-7.3 (-8.3 to -6.3)	<0.001	53.3 (51.8 to 54.7)	-6.7 (-8.2 to -5.1)	<0.001
	Hispanic	58.0 (56.7 to 59.3)	-2.1 (-3.5 to -0.8)	0.002	60.4 (58.5 to 62.4)	+0.5 (-1.5 to +2.5)	0.63
Palliative care counseling in last 180 days of life	NH White	22.0 (21.7 to 22.2)	Ref	Ref	22.1 (21.9 to 22.2)	Ref	Ref
	NH Black	24.5 (23.7 to 25.3)	+2.5 (+1.7 to +3.4)	<0.001	23.4 (22.3 to 24.5)	+1.3 (+0.2 to +2.5)	0.03
	Hispanic	22.9 (21.8 to 23.9)	+0.9 (-0.2 to +2.0)	0.10	23.1 (21.6 to 24.6)	+1.1 (-0.5 to +2.7)	0.17
Billed advance care planning before death	NH White	17.9 (17.7 to 18.1)	Ref	Ref	17.9 (17.8 to 18.0)	Ref	Ref
	NH Black	19.0 (18.3 to 19.8)	+1.1 (+0.3 to +1.9)	0.005	19.2 (18.2 to 20.2)	+1.3 (+0.2 to +2.4)	0.02
	Hispanic	18.5 (17.4 to 19.5)	+0.5 (-0.5 to +1.6)	0.32	18.3 (16.8 to 19.7)	+0.4 (-1.2 to +1.9)	0.64

Each model adjusted for age, sex, Medicaid coverage, nursing home residence, household income as estimated from residential zip code, comorbidities, year and month of death, and hospital referral region fixed effects. Model with physician fixed effects also adjusted for physician-level variation, effectively comparing beneficiaries treated by the same physician

Abbreviations: ED, emergency department; ICU, intensive care unit; NH, non-Hispanic; pp, percentage point; Ref: reference

© 2025 Oyeyemi DM et al. JAMA Health Forum.

eTable 4. End-of-Life Care Utilization and Processes for Dementia Decedents by Race and Ethnicity, Using E&M Claims from the Last 12 Months of Life to Attribute a Beneficiary to a Primary Care Physician

Outcomes	Race and ethnicity	Models without physician fixed effects			Models with physician fixed effects		
		Adjusted probability, % (95% CI)	Adjusted probability difference, pp (95% CI)	P-value	Adjusted probability, % (95% CI)	Adjusted probability difference, pp (95% CI)	P-value
ED visit in last 30 days of life	NH White	51.5 (51.3 to 51.7)	Ref	Ref	51.6 (51.5 to 51.7)	Ref	Ref
	NH Black	57.0 (56.4 to 57.7)	+5.5 (+4.9 to +6.2)	<0.001	56.8 (56.1 to 57.6)	+5.3 (+4.5 to +6.1)	<0.001
	Hispanic	57.0 (56.2 to 57.9)	+5.5 (+4.6 to +6.4)	<0.001	56.3 (55.2 to 57.4)	+4.7 (+3.6 to +5.8)	<0.001
Hospitalization in last 30 days of life	NH White	45.4 (45.2 to 45.6)	Ref	Ref	45.5 (45.4 to 45.6)	Ref	Ref
	NH Black	49.0 (48.4 to 49.7)	+3.6 (+3.0 to +4.3)	<0.001	48.6 (47.9 to 49.4)	+3.1 (+2.3 to +4.0)	<0.001
	Hispanic	51.0 (50.2 to 51.9)	+5.6 (+4.7 to +6.5)	<0.001	50.0 (48.9 to 51.1)	+4.5 (+3.4 to +5.6)	<0.001
ICU visit in last 30 days of life	NH White	22.6 (22.4 to 22.7)	Ref	Ref	22.7 (22.6 to 22.8)	Ref	Ref
	NH Black	26.8 (26.2 to 27.4)	+4.2 (+3.6 to +4.8)	<0.001	25.9 (25.2 to 26.6)	+3.2 (+2.5 to +3.9)	<0.001
	Hispanic	27.8 (27.0 to 28.6)	+5.2 (+4.4 to +6.1)	<0.001	26.6 (25.6 to 27.6)	+3.9 (+2.8 to +4.9)	<0.001
Mechanical ventilation or cardiopulmonary resuscitation in last 30 days of life	NH White	10.3 (10.2 to 10.4)	Ref	Ref	10.4 (10.3 to 10.4)	Ref	Ref
	NH Black	14.1 (13.6 to 14.6)	+3.8 (+3.3 to +4.3)	<0.001	13.4 (12.8 to 13.9)	+3.0 (+2.4 to +3.6)	<0.001
	Hispanic	13.4 (12.7 to 14.1)	+3.1 (+2.4 to +3.9)	<0.001	13.1 (12.2 to 13.9)	+2.7 (+1.8 to +3.6)	<0.001
Feeding tube placement in last 30 days of life	NH White	1.3 (1.2 to 1.3)	Ref	Ref	1.3 (1.2 to 1.3)	Ref	Ref
	NH Black	3.0 (2.7 to 3.2)	+1.7 (+1.4 to +1.9)	<0.001	2.8 (2.6 to 3.1)	+1.6 (+1.3 to +1.9)	<0.001
	Hispanic	1.6 (1.3 to 1.9)	+0.3	0.03	1.6 (1.2 to 1.9)	+0.3	0.10

© 2025 Oyeyemi DM et al. JAMA Health Forum.

			(+0.0 to +0.6)			(-0.1 to +0.7)	
Death in acute care hospital	NH White	15.6 (15.5 to 15.8)	Ref	Ref	15.8 (15.7 to 15.8)	Ref	Ref
	NH Black	18.9 (18.3 to 19.4)	+3.3 (+2.7 to +3.8)	<0.001	18.3 (17.6 to 18.9)	+2.5 (+1.8 to +3.2)	<0.001
	Hispanic	19.1 (18.3 to 19.8)	+3.5 (+2.7 to +4.2)	<0.001	17.5 (16.6 to 18.4)	+1.8 (+0.8 to +2.7)	<0.001
Hospice use in last 180 days of life	NH White	62.3 (62.2 to 62.5)	Ref	Ref	62.3 (62.2 to 62.4)	Ref	Ref
	NH Black	56.1 (55.4 to 56.7)	-6.3 (-7.0 to -5.6)	<0.001	56.2 (55.4 to 57.0)	-6.1 (-7.0 to -5.2)	<0.001
	Hispanic	60.3 (59.4 to 61.3)	-2.0 (-3.0 to -1.1)	<0.001	61.8 (60.7 to 63.0)	-0.4 (-1.6 to +0.8)	0.49
Palliative care counseling in last 180 days of life	NH White	19.4 (19.3 to 19.6)	Ref	Ref	19.6 (19.5 to 19.6)	Ref	Ref
	NH Black	22.4 (21.8 to 22.9)	+2.9 (+2.4 to +3.5)	<0.001	21.2 (20.6 to 21.8)	+1.7 (+1.0 to +2.3)	<0.001
	Hispanic	20.4 (19.7 to 21.1)	+0.9 (+0.2 to +1.7)	0.01	20.6 (19.7 to 21.4)	+1.0 (+0.1 to +1.9)	0.03
Billed advance care planning before death	NH White	16.0 (15.9 to 16.2)	Ref	Ref	16.1 (16.0 to 16.1)	Ref	Ref
	NH Black	17.6 (17.1 to 18.1)	+1.6 (+1.0 to +2.1)	<0.001	17.3 (16.8 to 17.9)	+1.3 (+0.6 to +1.9)	<0.001
	Hispanic	16.9 (16.2 to 17.7)	+0.9 (+0.1 to +1.6)	0.02	17.1 (16.3 to 17.9)	+1.0 (+0.2 to +1.9)	0.02

Each model adjusted for age, sex, Medicaid coverage, nursing home residence, household income as estimated from residential zip code, comorbidities, year and month of death, and hospital referral region fixed effects. Model with physician fixed effects also adjusted for physician-level variation, effectively comparing beneficiaries treated by the same physician

Abbreviations: ED, emergency department; ICU, intensive care unit; NH, non-Hispanic; pp, percentage point; Ref: reference

© 2025 Oyeyemi DM et al. JAMA Health Forum.

eTable 5. End-of-Life Care Utilization and Processes for Dementia Decedents by Race and Ethnicity, Using Claims from Office and Outpatient Settings to Define Palliative Care Counseling and Billed Advance Care Planning

Outcomes	Race and ethnicity	Models without physician fixed effects			Models with physician fixed effects		
		Adjusted probability, % (95% CI)	Adjusted probability difference, pp (95% CI)	P-value	Adjusted probability, % (95% CI)	Adjusted probability difference, pp (95% CI)	P-value
Palliative care counseling in last 180 days of life	NH White	1.3 (1.2 to 1.3)	Ref	Ref	1.3 (1.2 to 1.3)	Ref	Ref
	NH Black	1.4 (1.3 to 1.6)	+0.2 (+0.0 to +0.3)	0.045	1.3 (1.1 to 1.5)	+0.0 (-0.1 to +0.2)	0.66
	Hispanic	1.5 (1.3 to 1.8)	+0.3 (+0.1 to +0.5)	0.02	1.6 (1.3 to 1.8)	+0.3 (+0.0 to +0.6)	0.03
Billed advance care planning before death	NH White	4.4 (4.3 to 4.5)	Ref	Ref	4.3 (4.3 to 4.4)	Ref	Ref
	NH Black	4.0 (3.8 to 4.3)	-0.3 (-0.6 to -0.0)	0.03	4.4 (4.0 to 4.7)	+0.0 (-0.3 to +0.4)	0.92
	Hispanic	4.8 (4.4 to 5.3)	+0.5 (-0.0 to +0.9)	0.050	4.8 (4.3 to 5.3)	+0.4 (-0.1 to +0.9)	0.08

Each model adjusted for age, sex, Medicaid coverage, nursing home residence, household income as estimated from residential zip code, comorbidities, year and month of death, and hospital referral region fixed effects. Model with physician fixed effects also adjusted for physician-level variation, effectively comparing beneficiaries treated by the same physician
Abbreviations: NH, non-Hispanic; pp, percentage point; Ref: reference

© 2025 Oyeyemi DM et al. JAMA Health Forum.

eTable 6. End-of-Life Care Utilization and Processes for Dementia Decedents with Annual Wellness Visits by Race and Ethnicity

Outcomes	Race and ethnicity	Models without physician fixed effects			Models with physician fixed effects		
		Adjusted probability, % (95% CI)	Adjusted probability difference, pp (95% CI)	P-value	Adjusted probability, % (95% CI)	Adjusted probability difference, pp (95% CI)	P-value
ED visit in last 30 days of life	NH White	60.4 (60.0 to 60.8)	Ref	Ref	60.4 (60.2 to 60.6)	Ref	Ref
	NH Black	65.9 (64.4 to 67.3)	+5.5 (+4.0 to +7.0)	<0.001	66.3 (64.1 to 68.5)	+5.9 (+3.5 to +8.3)	<0.001
	Hispanic	64.2 (62.2 to 66.3)	+3.9 (+1.8 to +5.9)	<0.001	63.5 (60.2 to 66.8)	+3.1 (-0.4 to +6.5)	0.08
Hospitalization in last 30 days of life	NH White	54.1 (53.6 to 54.5)	Ref	Ref	54.2 (53.9 to 54.4)	Ref	Ref
	NH Black	57.6 (56.1 to 59.1)	+3.5 (+2.0 to +5.1)	<0.001	57.3 (55.0 to 59.5)	+3.1 (+0.7 to +5.5)	0.01
	Hispanic	58.9 (56.9 to 61.0)	+4.9 (+2.8 to +7.0)	<0.001	57.2 (53.8 to 60.5)	+3.0 (-0.5 to +6.5)	0.09
ICU visit in last 30 days of life	NH White	28.2 (27.8 to 28.6)	Ref	Ref	28.2 (28.0 to 28.4)	Ref	Ref
	NH Black	30.9 (29.5 to 32.4)	+2.8 (+1.2 to +4.3)	<0.001	31.1 (29.0 to 33.3)	+2.9 (+0.6 to +5.3)	0.01
	Hispanic	32.4 (30.4 to 34.4)	+4.2 (+2.1 to +6.3)	<0.001	31.5 (28.3 to 34.7)	+3.3 (-0.0 to +6.7)	0.053
Mechanical ventilation or cardiopulmonary resuscitation in last 30 days of life	NH White	13.1 (12.8 to 13.4)	Ref	Ref	13.2 (13.0 to 13.3)	Ref	Ref
	NH Black	16.6 (15.4 to 17.9)	+3.6 (+2.3 to +4.9)	<0.001	15.9 (14.1 to 17.7)	+2.8 (+0.8 to +4.7)	0.005
	Hispanic	15.7 (14.0 to 17.4)	+2.7 (+0.9 to +4.4)	0.003	14.8 (12.2 to 17.4)	+1.6 (-1.1 to +4.3)	0.23
Feeding tube placement in last 30 days of life	NH White	1.5 (1.3 to 1.6)	Ref	Ref	1.5 (1.4 to 1.5)	Ref	Ref
	NH Black	3.3 (2.7 to 3.9)	+1.9 (+1.2 to +2.5)	<0.001	3.1 (2.3 to 4.0)	+1.6 (+0.7 to +2.6)	<0.001
	Hispanic	2.2 (1.5 to 2.8)	+0.7 (+0.0 to +1.4)	0.045	2.2 (1.2 to 3.1)	+0.7 (-0.3 to +1.7)	0.16

© 2025 Oyeyemi DM et al. JAMA Health Forum.

Death in acute care hospital	NH White	18.7 (18.4 to 19.1)	Ref	Ref	18.9 (18.7 to 19.0)	Ref	Ref
	NH Black	21.9 (20.6 to 23.3)	+3.2 (+1.8 to +4.6)	<0.001	21.2 (19.2 to 23.1)	+2.3 (+0.2 to +4.4)	0.03
	Hispanic	24.0 (22.2 to 25.9)	+5.3 (+3.4 to +7.2)	<0.001	22.5 (19.7 to 25.3)	+3.6 (+0.7 to +6.5)	0.01
Hospice use in last 180 days of life	NH White	62.6 (62.2 to 63.1)	Ref	Ref	62.5 (62.3 to 62.8)	Ref	Ref
	NH Black	54.6 (53.0 to 56.2)	-8.0 (-9.7 to -6.4)	<0.001	54.8 (52.3 to 57.2)	-7.8 (-10.4 to -5.1)	<0.001
	Hispanic	58.3 (56.2 to 60.5)	-4.3 (-6.5 to -2.1)	<0.001	60.7 (57.5 to 64.0)	-1.8 (-5.2 to +1.6)	0.31
Palliative care counseling in last 180 days of life	NH White	23.2 (22.9 to 23.6)	Ref	Ref	23.3 (23.1 to 23.5)	Ref	Ref
	NH Black	26.2 (24.8 to 27.6)	+3.0 (+1.5 to +4.4)	<0.001	25.7 (23.7 to 27.8)	+2.4 (+0.2 to +4.6)	0.03
	Hispanic	23.6 (21.8 to 25.3)	+0.3 (-1.5 to +2.2)	0.71	22.8 (20.1 to 25.5)	-0.5 (-3.4 to +2.3)	0.71
Billed advance care planning before death	NH White	26.7 (26.3 to 27.0)	Ref	Ref	26.9 (26.7 to 27.1)	Ref	Ref
	NH Black	29.1 (27.7 to 30.6)	+2.5 (+0.9 to +4.0)	0.002	27.6 (25.5 to 29.7)	+0.7 (-1.5 to +2.9)	0.54
	Hispanic	30.0 (28.0 to 32.0)	+3.3 (+1.3 to +5.4)	0.002	27.5 (24.7 to 30.3)	+0.7 (-2.3 to +3.6)	0.66

Each model adjusted for age, sex, Medicaid coverage, nursing home residence, household income as estimated from residential zip code, comorbidities, year and month of death, and hospital referral region fixed effects. Model with physician fixed effects also adjusted for physician-level variation, effectively comparing beneficiaries treated by the same physician

Abbreviations: ED, emergency department; ICU, intensive care unit; NH, non-Hispanic; pp, percentage point; Ref: reference

© 2025 Oyeyemi DM et al. JAMA Health Forum.

eTable 7. Changes in End-of-Life Care Utilization and Processes for Dementia Decedents by Race and Ethnicity, 2016-2019

Outcomes	Race and ethnicity	Adjusted proportion in 2016, %	Annual percentage-point change	P-for-trend	P-for-interaction
ED visit in last 30 days of life	NH White	54.0	-0.7	<.001	Reference
	NH Black	60.7	-0.8	.01	.85
	Hispanic	60.9	-1.4	.001	.09
Hospitalization in last 30 days of life	NH White	47.9	-0.8	<.001	Reference
	NH Black	52.8	-0.7	.02	.77
	Hispanic	54.5	-1.2	.003	.29
ICU visit in last 30 days of life	NH White	23.4	-0.1	.46	Reference
	NH Black	27.5	+0.2	.45	.35
	Hispanic	29.4	+0.1	.77	.67
Mechanical ventilation or cardiopulmonary resuscitation in last 30 days of life	NH White	10.4	+0.1	.09	Reference
	NH Black	15.7	-0.3	.25	.13
	Hispanic	13.2	+0.5	.12	.21
Feeding tube placement in last 30 days of life	NH White	1.5	-0.1	<0.001	Reference
	NH Black	3.2	-0.3	.01	.10
	Hispanic	1.9	-0.2	.20	.58
Death in acute care hospital	NH White	16.7	-0.5	<.001	Reference
	NH Black	21.0	-0.5	.06	.92
	Hispanic	20.1	-0.7	.05	.52
Hospice use in last 180 days of life	NH White	59.1	+1.4	<.001	Reference
	NH Black	52.9	+1.1	.001	.41
	Hispanic	57.3	+1.5	<.001	.71
Palliative care counseling in last 180 days of life	NH White	15.7	+2.4	<.001	Reference
	NH Black	18.3	+2.9	<.001	.04
	Hispanic	17.2	+2.2	<.001	.54
Billed advance care planning before death	NH White	4.8	+6.9	<.001	Reference
	NH Black	3.7	+8.7	<.001	<.001
	Hispanic	2.8	+8.0	<.001	.001

Each model adjusted for age, sex, Medicaid coverage, nursing home residence, household income as estimated from residential zip code, comorbidities, year and month of death, and hospital referral region fixed effects.
Abbreviations: ED, emergency department; ICU, intensive care unit; NH, non-Hispanic

© 2025 Oyeyemi DM et al. JAMA Health Forum.

eReferences

1. McWilliams JM, Hatfield LA, Landon BE, Hamed P, Chernew ME. Medicare Spending after 3 Years of the Medicare Shared Savings Program. *N Engl J Med*. 2018;379(12):1139-1149. doi:10.1056/NEJMsa1803388

2. McWilliams JM, Gilstrap LG, Stevenson DG, Chernew ME, Huskamp HA, Grabowski DC. Changes in Postacute Care in the Medicare Shared Savings Program. *JAMA Intern Med*. 2017;177(4):518-526. doi:10.1001/jamainternmed.2016.9115

3. McWilliams JM, Hatfield LA, Chernew ME, Landon BE, Schwartz AL. Early Performance of Accountable Care Organizations in Medicare. *N Engl J Med*. 2016;374(24):2357-2366. doi:10.1056/NEJMsa1600142

4. Research Data Assistance Center. Research Triangle Institute (RTI) race code. Accessed July 24, 2024. https://resdac.org/cms-data/variables/medicare-research-triangle-institute-rti-race-code

5. Eicheldinger C, Bonito A. More accurate racial and ethnic codes for Medicare administrative data. *Health Care Financ Rev*. 2008;29(3):27-42.

© 2025 Oyeyemi DM et al. JAMA Health Forum.
