# Supplementary material for: End-of-Life Care for Older Adults With Dementia by Race and Ethnicity and Physicians’ Role
Source: JAMA Health Forum. 2025 Nov 14;6(11):e254235. doi: 10.1001/jamahealthforum.2025.4235 (PMC12619101; doi:10.1001/jamahealthforum.2025.4235)
Supplement: Supplement 2. — Data Sharing Statement [file jamahealthforum-e254235-s002.pdf]

Data Sharing Statement

Oyeyemi. End-of-Life Care for Older Adults With Dementia by Race and Ethnicity and Physicians’ Role. *JAMA Health Forum*. Published November 14, 2025. doi:10.1001/jamahealthforum.2025.4235

Data
Data available: No

Additional Information
Explanation for why data not available: Given our Medicare data contains (encrypted) individual identifiers of Medicare beneficiaries, the Centers for Medicare & Medicaid Services (CMS) prohibits us from sharing the data with other researchers or entities. Medicare data can be purchased independently from CMS by any researcher with a valid research proposal.
